# Detection of *Mycobacterium bovis* in nasal swabs from communal goats (*Capra hircus*) in rural KwaZulu-Natal, South Africa

**DOI:** 10.3389/fmicb.2024.1349163

**Published:** 2024-02-14

**Authors:** Deborah M. Cooke, Charlene Clarke, Tanya J. Kerr, Robin M. Warren, Carmel Witte, Michele A. Miller, Wynand J. Goosen

**Affiliations:** ^1^Division of Molecular Biology and Human Genetics, Faculty of Medicine and Health Sciences, DSI-NRF Centre of Excellence for Biomedical Tuberculosis Research, South African Medical Research Council Centre for Tuberculosis Research, Stellenbosch University, Stellenbosch, South Africa; ^2^Faculty of Natural and Agricultural Sciences, Department of Zoology and Entomology, University of Pretoria, Pretoria, South Africa; ^3^The Center for Wildlife Studies, South Freeport, ME, United States

**Keywords:** animal tuberculosis, culture-independent detection, GeneXpert® MTB/RIF Ultra, *Capra hircus*, *hsp65*, *rpoB*, Ion Torrent Genexus sequencing, *Mycobacterium bovis*

## Abstract

Animal tuberculosis, caused by *Mycobacterium bovis*, presents a significant threat to both livestock industries and public health. *Mycobacterium bovis* tests rely on detecting antigen specific immune responses, which can be influenced by exposure to non-tuberculous mycobacteria, test technique, and duration and severity of infection. Despite advancements in direct *M. bovis* detection, mycobacterial culture remains the primary diagnostic standard. Recent efforts have explored culture-independent PCR-based methods for identifying mycobacterial DNA in respiratory samples. This study aimed to detect *M. bovis* in nasal swabs from goats (*Capra hircus*) cohabiting with *M. bovis*-infected cattle in KwaZulu-Natal, South Africa. Nasal swabs were collected from 137 communal goats exposed to *M. bovis*-positive cattle and 20 goats from a commercial dairy herd without *M. bovis* history. Swabs were divided into three aliquots for analysis. The first underwent GeneXpert® MTB/RIF Ultra assay (Ultra) screening. DNA from the second underwent mycobacterial genus-specific PCR and Sanger sequencing, while the third underwent mycobacterial culture followed by PCR and sequencing. Deep sequencing identified *M. bovis* DNA in selected Ultra-positive swabs, confirmed by region-of-difference (RD) PCR. Despite no other evidence of *M. bovis* infection, viable *M. bovis* was cultured from three communal goat swabs, confirmed by PCR and sequencing. Deep sequencing of DNA directly from swabs identified *M. bovis* in the same culture-positive swabs and eight additional communal goats. No *M. bovis* was found in commercial dairy goats, but various NTM species were detected. This highlights the risk of *M. bovis* exposure or infection in goats sharing pastures with infected cattle. Rapid Ultra screening shows promise for selecting goats for further *M. bovis* testing. These techniques may enhance *M. bovis* detection in paucibacillary samples and serve as valuable research tools.

## 1 Introduction

*Mycobacterium bovis* (*M. bovis*), a member of the *Mycobacterium tuberculosis* complex (MTBC), is the causative agent of animal tuberculosis (Borham et al., 2022). Although cattle are considered the primary host, *M. bovis* is known to have the widest host range of all members of the MTBC, with the ability to cause disease in domestic animals, wildlife, and humans (Mostowy et al., 2005; Palmer et al., 2012). The World Organization for Animal Health (WOAH) lists animal tuberculosis (bTB) as a notifiable disease (World Organization for Animal Health, 2023) and many developed countries have programs to manage and control this disease in livestock, primarily to prevent spread to humans (Reviriego Gordejo and Vermeersch, 2006; Palmer and Waters, 2011; World Health Organization, 2017; More, 2019).

Tuberculosis in domestic goats (*Capra hircus*) is mostly attributed to *Mycobacterium bovis* (*M. bovis*) and *M. caprae*, which are capable of infecting other animals, including humans (Rodríguez et al., 2009; Pesciaroli et al., 2014; Bezos et al., 2015). Zoonotic TB remains a considerable global challenge; in 2019, an estimated 140,000 new cases and 11,400 deaths were reported (World Health Organization, 2020). In South Africa, *M. bovis* is endemic in communal cattle and some wildlife populations with TB control programs predominantly focusing on cattle (Renwick et al., 2007; Arnot and Michel, 2020). The epidemiology and diagnosis of goat TB are similar to cattle, which are both natural hosts (Quintas et al., 2010; Pesciaroli et al., 2014). Although there are published reports of *M. bovis* testing of goats in other countries (Quintas et al., 2010), *M. bovis* in domestic goats has not been thoroughly investigated in South Africa (Nyoni, 2019). The traditional approach to keeping livestock in South African communities involves goats sharing communal pastures and water sources with cattle, and occasionally with wildlife; therefore, the lack of surveillance may lead to under-recognition of goats as a potential source of *M. bovis* spread to cattle or other livestock as well as humans and wildlife.

Tests that accurately identify *M. bovis* infected individuals and herds are the foundation of bTB control programs. Most *M. bovis* diagnostic tests for livestock rely on detecting host antigen-specific cell-mediated immune (CMI) responses to mycobacterial antigens, typically the *in vivo* tuberculin skin test (Welsh et al., 2005; Bernitz et al., 2021). Currently, the official South African (SA) guidelines for TB testing in livestock and African buffaloes (*Syncerus caffer*) advocate the use of the single intradermal comparative tuberculin test (SICTT; Department of Agriculture, Forestry and Fisheries of SA, 2016; Department of Agriculture, Forestry and Fisheries of SA, 2017; Department of Agriculture, Land Reform and Rural Development, 2018; Arnot and Michel, 2020). However, the interpretation of the SICTT can be confounded by several factors including exposure to environmental non-tuberculous mycobacteria (NTM), which may cause cross-reactivity (Michel et al., 2011). Since NTMs are ubiquitous in the environment (Falkinham, 2021), with a high diversity occurring in SA (Gcebe et al., 2013), their presence may lead to *M. bovis* false-positive reactions in tested animals. This, in turn, may cause unnecessary expenses due to additional testing, loss of income, and loss of animals (Vordermeier et al., 2007; Bolaños et al., 2017). This is especially problematic for rural farmers in SA, who depend on small-scale livestock farming as a source of income (Sichewo et al., 2020).

While the SICTT is routinely used for antemortem screening, definitive diagnosis of bTB is based on the direct detection of *M. bovis* from animal tissue samples using mycobacterial culture, followed by speciation using region-of-difference (RD) PCRs (Warren et al., 2006; Bernitz et al., 2021). However, conventional mycobacterial culture has suboptimal performance, is slow, laborious (Ghodbane et al., 2014), introduces *in vitro* bacterial selection pressure, and may lead to false negative results (especially with paucibacillary samples) due to harsh sample decontamination steps (de Boer et al., 2002). Although technical advances have improved direct detection of pathogenic *Mycobacteria* spp., most applications still heavily rely on culture to obtain sufficient organisms to confirm infection (Bernitz et al., 2021). Therefore, it is important to continuously explore new methods, in addition to culture, for the direct detection of *M. bovis* infected livestock.

Recently, culture-independent PCR-based sequencing methods have been investigated for direct detection and identification of important mycobacterial organisms in antemortem respiratory samples and postmortem tissues (Clarke et al., 2022a; Goosen et al., 2022a). Studies have shown that the GeneXpert® MTB/RIF Ultra assay (Ultra) can detect MTBC DNA in animal samples and provides a rapid sensitive screening test (Goosen et al., 2020; Clarke et al., 2021). Furthermore, conventional, and real-time PCR assays, followed by amplicon sequencing, have also shown promise for detecting and characterizing both MTBC and NTM species present in cultures, as well as directly from raw specimens (Warren et al., 2006; Deggim-Messmer et al., 2016; Jung et al., 2016; Goosen et al., 2020, 2022a; Clarke et al., 2022a,b). Amplicons from these PCRs can be used for targeted next generation sequencing, which facilitates the accurate detection and characterization of multiple mycobacterial species present, especially in paucibacillary oronasal swabs, other respiratory samples, as well as fecal samples (Adékambi et al., 2003; Lee et al., 2020; Goosen et al., 2022a). Combining culture with these techniques will enable confirmation of infection in animals with positive host CMI test results.

Culture-independent detection of pathogenic mycobacterial species using extracted DNA can enhance individual diagnosis, identify infected herds, and improve disease management, especially when samples for culture cannot be transported due to sample movement restrictions, or where there is no laboratory capacity for mycobacterial culture. In SA, this is especially relevant when testing rural livestock in areas where the presence of controlled diseases, such as Foot and Mouth Disease (FMD), restrict movement of animals and samples, unless they are heat-inactivated (Brückner et al., 2002). Therefore, the aim of this study was to perform MTBC- and *Mycobacterium* genus-specific PCRs and sequencing, using DNA extracted directly from swabs and from swab cultures, to determine the presence and species of MTBC in goat respiratory samples.

## 2 Materials and methods

### 2.1 Ethics

The Stellenbosch University Animal Care and Use Committee granted ethical approval for this project (ACU-2020-14560) and Section 20 approval was issued by the Department of Agriculture, Land Reform, and Rural Development (DALRRD) (12/11/1/7/2 (16045S)). Consent was obtained from all goat owners prior to testing.

### 2.2 Goat nasal swab collection, processing, and mycobacterial culture

**Figure 1:**
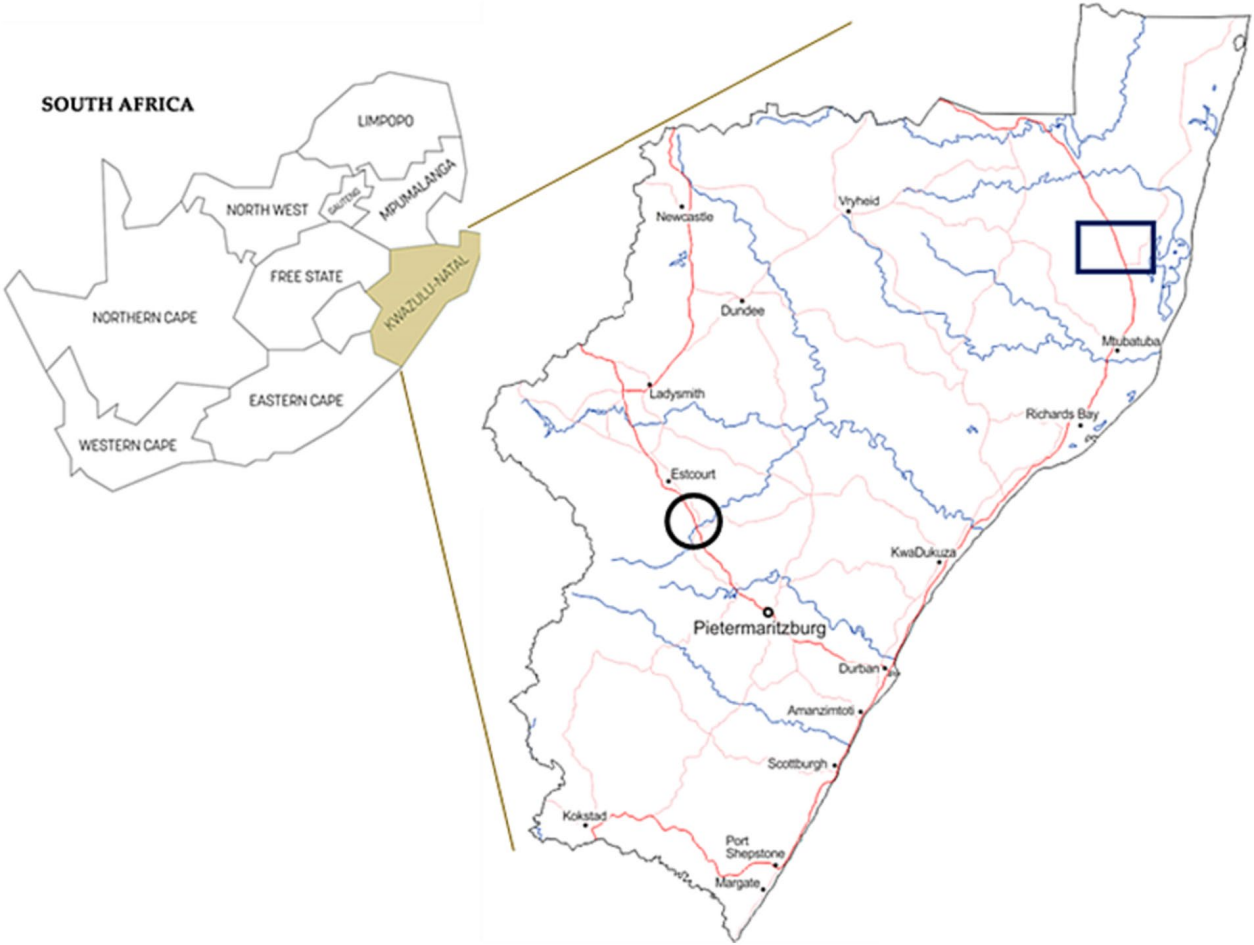
Map of South Africa with province of KwaZulu-Natal shown in insert. Sampling area of communal domestic goat herds are indicated by a black square. Black circles show the location of the commercial dairy goat herd.

In 2019, nasal swabs were collected from 157 goats (*Capra hircus*) from the KwaZulu-Natal midlands, SA. This included opportunistic sampling from 137 communal domestic goats in an area with confirmed *M. bovis* infected cattle (Umkhanyakude district of Northern Zululand, KwaZulu-Natal) and sampling from 20 goats in a closed commercial dairy herd consisting of stud Saanen goats with no known exposure to *M. bovis*, a 20-year long history of negative annual SICCT tests, and a high level of management and biosecurity practices, as previously described (Cooke et al., 2023; Figure 1). Nasal swabs were taken using sterile OmniSwabs (Whatman®, Qiagen, Germantown, MD, United States). Swab heads were placed directly into cryovials containing ~1.5 mL sterile saline solution, transported with ice bricks in a cooler box, and subsequently frozen at −80°C within 8 h of collection, before being transported to Stellenbosch University for further downstream processing.

**Figure 2:**
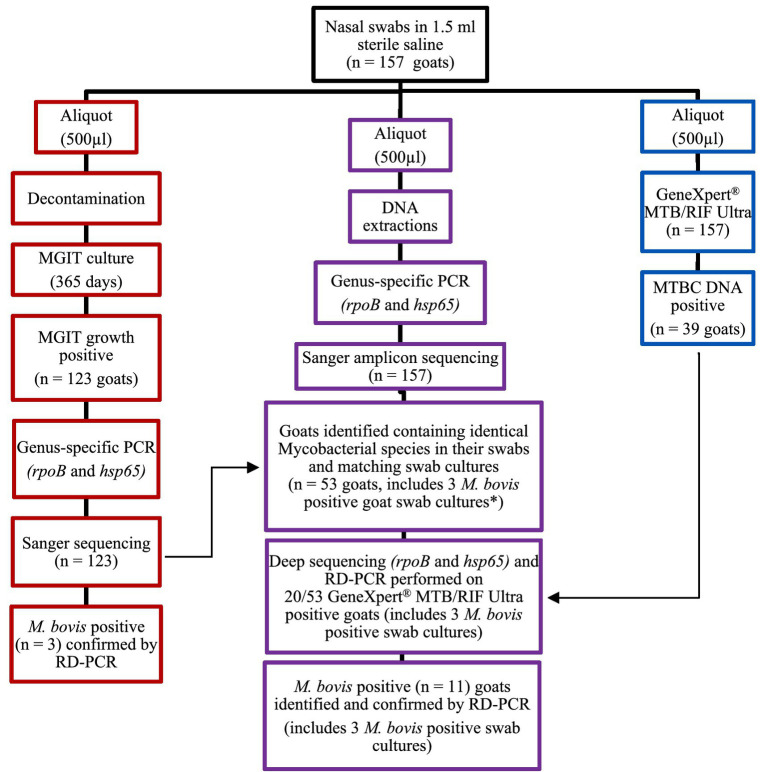
Flow chart for goat nasal swab (*n* = 157) sample processing for PCR testing and mycobacterial identification. The red section (left) shows the process of aliquot 3 for mycobacterial culture and PCR; the purple section (middle) shows the process followed for the raw swab samples (aliquot 2); and the blue section (right) shows how aliquot 1 was processed directly for MTBC DNA detection using the GeneXpert® MTB/RIF Ultra assay. *Three *Mycobacterium bovis* culture-positive goats also had *M. bovis* DNA detected using culture-independent processes for the raw swabs; these were goats (C139, F91, and N112).

After transport of frozen nasal swabs, all samples (*n* = 157) were split into three aliquots of ~500 μL each in a biosafety level 3 (BSL-3) facility (Figure 2). One aliquot was processed directly for MTBC DNA detection using the GeneXpert® MTB/RIF Ultra assay (Cepheid, Sunnyvale, CA, United States), as previously described (Goosen et al., 2020). A second aliquot underwent DNA extraction, using the DNeasy Blood and Tissue kit (Qiagen, Hilden, Germany) as instructed by the manufacturer. The extracted DNA was used for *Mycobacterium* genus-specific PCRs (*hsp65* and *rpoB*), subsequent *Mycobacteria* spp. identification by amplicon sequencing using Sanger and a NGS platform as well as RD-PCR speciation of all MTBC DNA positive samples, as described below (Goosen et al., 2022a). The third aliquot was decontaminated by using a 1:1 volume of MycoPrep to sample volume, incubated for 15 min and then neutralized with sterile PO4 buffer (at the same volume of sample plus MycoPrep). The sample was then shaken to produce a homogeneous solution and then centrifuged for 15 min at 2,000× g for MGIT inoculation, after which, the supernatant was aspirated off, down to a remaining 1 mL buffer volume just above the pellet. The pellet was then thoroughly reconstituted and transferred to Mycobacterial Growth Indicator Tubes (MGIT) and incubated in the BACTECTM MGITTM 960 TB system (Becton Dickinson, Franklin Lakes, NJ, United States), as previously described, with a minor modification (Goosen et al., 2022b).

Briefly, all MGIT tubes, including two uninoculated MGIT tubes designated as negative controls, underwent aseptic replenishment with 1.5 mL fresh media sourced from new MGIT tubes (Becton Dickinson, United States). This procedure was conducted in batches of 5 MGITs at a time within a Class II Biological Safety Cabinet located in a BSL-3 facility every 56 days, spanning up to 365 days of incubation. At each 56-day interval, RD-PCRs were performed on 1.5 mL boiled MGIT homogenates (20 min at 98°C followed by centrifugation at 2,000× *g* for 20 min to ensure maximum release of DNA and to reduce the possibility of discarding some paucibacillary bacilli) from all MGIT tubes to check for the presence of MTBC DNA. Only at the final 365-day time point, DNA was extracted from 1.5 mL aliquots from all MGIT cultures, followed by a genus-specific PCR, subsequent Sanger amplicon sequencing and MTBC speciation by RD-PCR of all samples identified as containing MTBC DNA following amplicon sequencing, as described below. Furthermore, spoligotyping were performed on all *M. bovis*-positive MGIT samples (confirmed by RD-PCR) following an established protocol as described by Kamerbeek et al. (1997). It is noteworthy that the MGIT negative control tubes consistently maintained a culture-negative status throughout the entire duration of this process.

### 2.3 Culture-independent screening of raw goat nasal swab samples for MTBC DNA using GeneXpert® MTB/RIF Ultra

The Ultra assay (Cepheid) was performed on all raw nasal swab aliquots (*n* = 157; Goosen et al., 2020). Briefly, Ultra sample lysis reagent was added to the swab sample aliquot at a ratio of 2:1, thoroughly vortexed for 10 s, incubated at room temperature for 10 min, vortexed for 10 s, and incubated for a final 5 min at room temperature in a BSL-3 laboratory. Thereafter, the solution was transferred into the sample chamber of the Ultra cartridge. Samples were analyzed for the presence of MTBC DNA using the automated GeneXpert module PCR system (Cepheid). The read-out of the Ultra assay was recorded as MTB detected high, medium, low, very low, MTB trace detected, or MTB not detected (Goosen et al., 2020). The “MTB not detected” read-out was regarded as an Ultra negative result (no MTBC DNA present) and all other read-outs were considered a positive Ultra result.

### 2.4 Nucleic acid amplification tests and amplicon sequencing for *mycobacteria* spp. detection and identification

*Mycobacterium* genus-specific *rpoB* (764 bp) and *hsp65* (436 bp) Q5 HiFi Taq (New England Biolabs, Ipswich, MA, United States) PCRs were performed to detect the presence of any *Mycobacteria* spp. These PCRs used DNA extracted from (a) 1.5 mL boiled aliquots from MGIT cultures (*n* = 157) after 365 days and (b) a 500 μL aliquot directly from raw nasal swabs (*n* = 157), as previously described (Clarke et al., 2022b). The PCR amplicons for *rpoB* and *hsp65* (referred to as genus-specific PCR) from cultures were pooled for each goat and sent to the Central Analytical Facility (CAF, Stellenbosch University, Stellenbosch, SA) for Sanger sequencing. Similarly, genus-specific PCR amplicons (*hsp65* and *rpoB*) using DNA extracted from raw swabs (b) were also pooled for each goat and further deep sequenced as a separate sample for a select few animals using the Ion S5™ next generation sequencing platform (Thermo Fisher, Waltham, MA, United States) at CAF.

Briefly, genus-specific amplicon pools (*hsp65* and *rpoB*) of each animal’s raw swabs were selected to undergo deep sequencing if they had Ultra positive results and if they had concordant *Mycobacteria* spp. results identified through Sanger sequencing between raw swabs and swab cultures. Furthermore, all MTBC positive results, as indicated by Sanger and/or deep sequencing, were further speciated using RD-PCR to confirm the presence or absence of *M. bovis,* as previously described (Warren et al., 2006). Controls included: (1) DNA extraction controls, and (2) PCR amplification controls (positive and negative). All controls were included during each PCR and subsequent sequencing events.

For all Sanger sequences generated, mycobacterial species identification was performed by NCBI BLAST analysis using a species identity match threshold ≥99 and 100% coverage as previously described (Clarke et al., 2022b). For Ion Torrent sequencing, Flow space calibration and BaseCaller analyses were performed using default analysis parameters in the Torrent Suite Version 5.16.1 software (Thermo Fisher). Deep sequences generated by the Ion Torrent platform reference-free species level identification, down to taxonomic level 7, were identified using QIIME2’s database and QIIME2view,^1^ as previously described (Shi et al., 2019; Figure 2).

### 2.5 Data analyses

Frequency distributions of identified mycobacterial species in swab samples, determined by Sanger sequencing, were grouped, and reported by the number of goats tested. The Ion Torrent amplicon deep sequencing results were reported as the percentage of high-quality sequenced reads assigned to a specific *Mycobacteria* spp.; this provided a description of the relative abundance of identified mycobacteria in the polymicrobial samples (goat nasal swabs; Deurenberg et al., 2017).

## 3 Results

**Figure 3:**
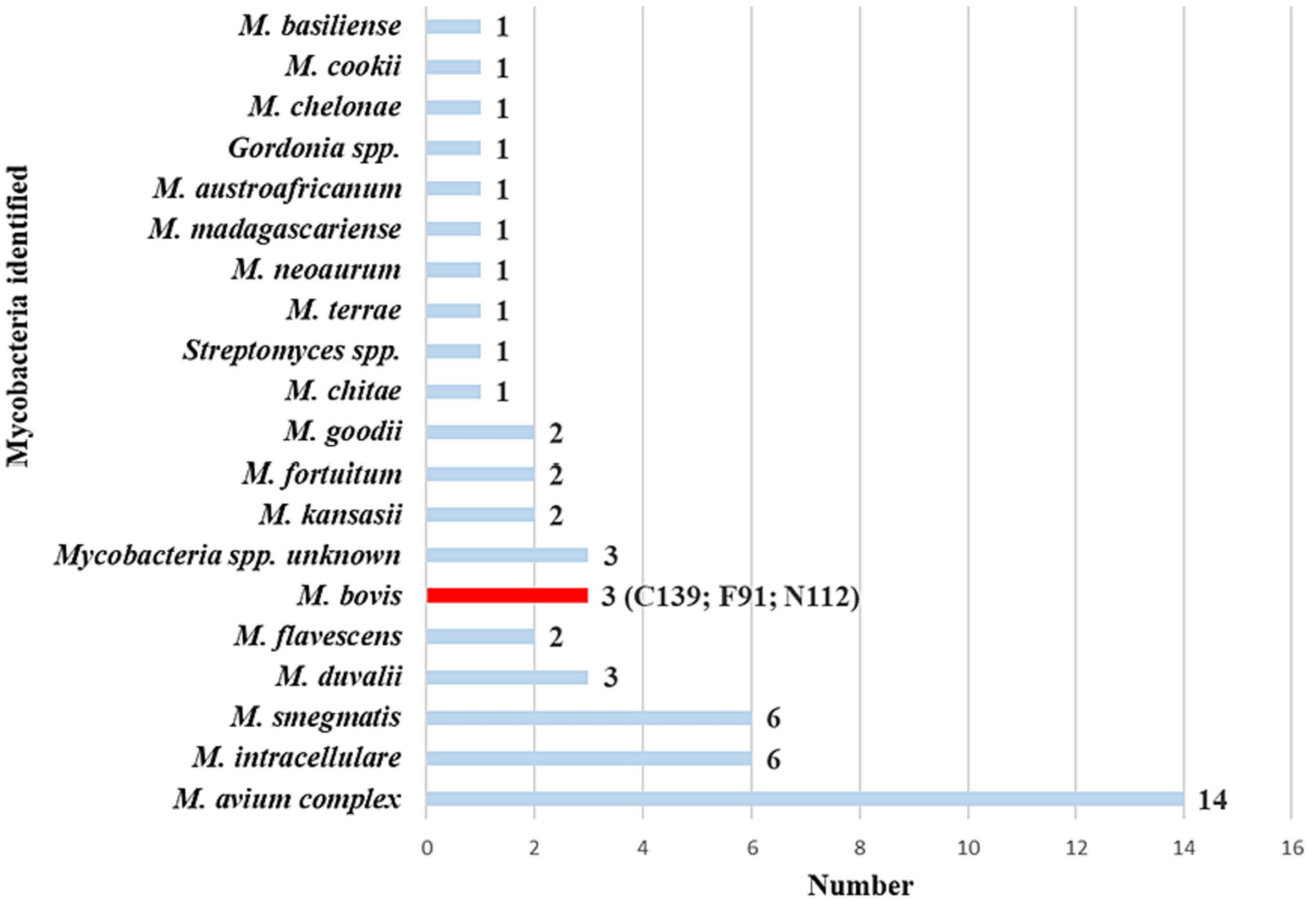
*Mycobacteria* spp. identified from 53 goat nasal swabs using genus-specific PCR targeting *hsp65* and *rpoB*. The PCR amplicons from the two targets were pooled from raw swab DNA and matching swab culture samples, then subjected to Sanger sequencing. There was complete concordance of identified mycobacterial species between both gene targets and sample types for each animal. This provided confidence in the results, leading to the selection of these animals as the focus group for further investigation. Subsequently, samples were further speciated using region-of-difference (RD) PCR to confirm the presence or absence of *Mycobacterium bovis*. The number of goats with each species of *Mycobacterium* is reported next to each species name. Goat IDs for the three *M. bovis* positive goats were C139, F91, and N112.

Of the 157 swab samples processed for mycobacterial culture, MGIT growth was detected in 123 (78%). Based on genus-specific PCR amplicon Sanger results, matching *Mycobacteria* spp. were identified in paired raw swab DNA and swab cultures from 53 goats (Figure 3; Supplementary Table 1; i.e., complete concordance). Moreover, raw swab aliquots from all goats were screened for MTBC DNA with the GeneXpert® MTB/RIF Ultra. Of the 53 goats of interest, 20 (38%) had positive results for MTBC DNA using the Ultra. This included one swab from a commercial goat herd (Supplementary Table 2).

**Figure 4:**
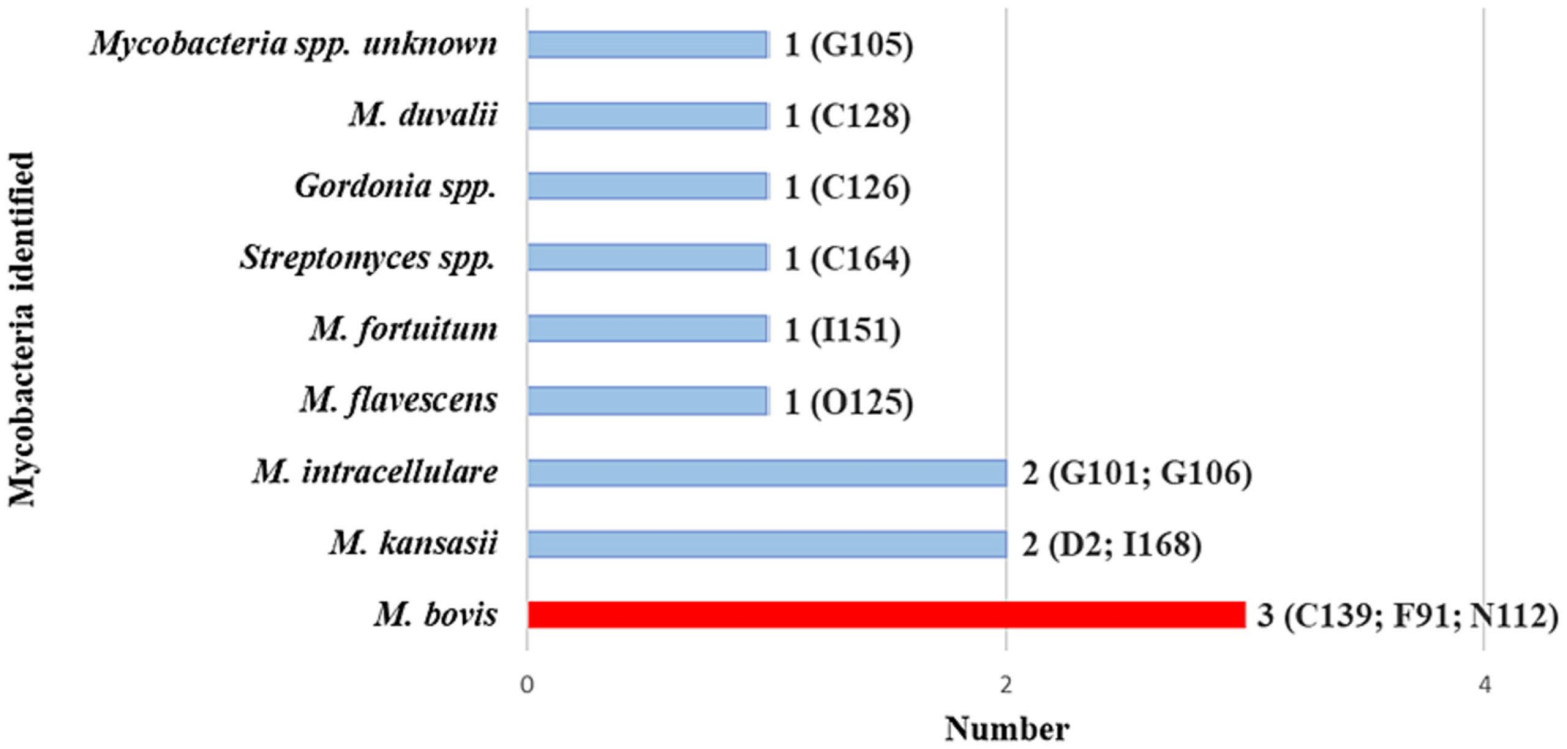
Mycobacterial species identified by Sanger sequencing of pooled *hsp*65 and *rpoB* amplicons generated from DNA extracted from culture and raw swabs in the 20 GeneXpert® MTB/RIF Ultra positive goats. These goats are a subset of the 53 with the same *Mycobacteria* spp. in both the raw samples and cultured samples, as per Sanger sequencing results (shown in Figure 3). Individual goat ID and number of goats with a specific *Mycobacterium* spp. are shown. Goat IDs for the three *Mycobacterium bovis*-positive goats were C139, F91, and N112.

Swab samples from the 20 goats with positive Ultra results were further evaluated by performing a genus-specific PCR with Sanger sequencing and RD-PCR to confirm and speciate any MTBC present. Results showed that 3 goats had *M. bovis* DNA in paired cultured and raw swab samples. Unfortunately, further speciation by spoligotyping was unsuccessful for all *M. bovis-*positive MGIT samples. All three goats were from communal herds. Figure 4 shows the diversity of all mycobacterial species identified in the 20 Ultra positive goats.

**Figure 5:**
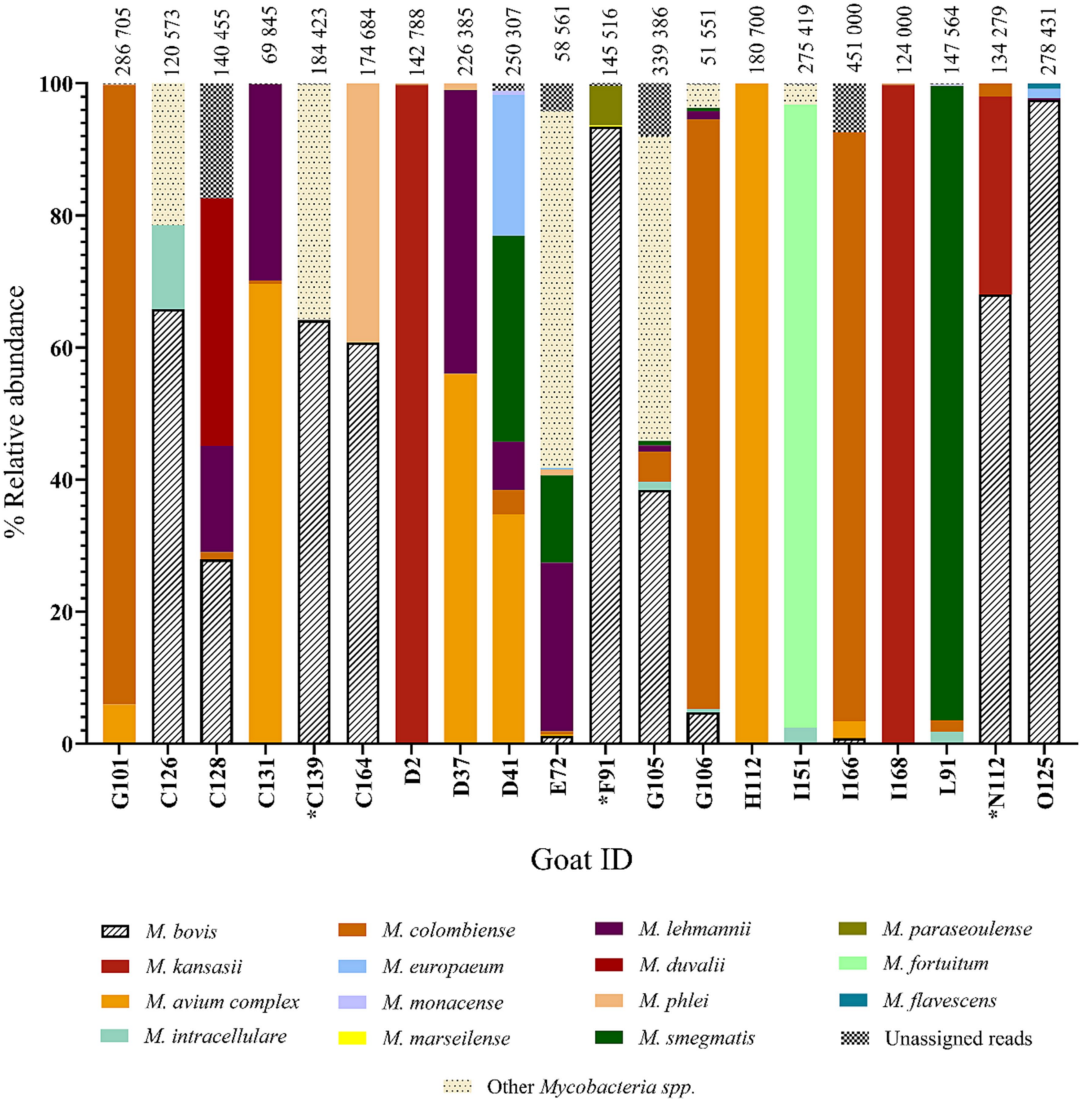
Relative abundance of *Mycobacteria* spp. by individual goat ID (*n* = 20). Data represent the amplicon deep sequencing read for *rpoB* and *hsp65* combined that aligned to various *Mycobacteria* spp. identified from the raw swab DNA. The 20 goats shown here are also represented in Figure 4 and include all animals that were GeneXpert® MTB/RIF Ultra positive that had the same *Mycobacteria* spp. Sanger sequencing results for their raw swab DNA and the matching swab culture. *Mycobacterium bovis was detected by all three approaches (*M. bovis* DNA discovered by genus-specific PCR and Sanger sequencing of both culture and raw samples, as well as by deep sequencing of amplicon targets, Goat IDs: C139, F91, and N112).

In addition, the amplicon pools derived from the 20 raw swab samples, were subjected to PCR amplicon deep sequencing. *Mycobacterium bovis* DNA was identified in 11 out of 20 goat swab samples (Supplementary Table 1). The positive group included the 3 goats (goat IDs: C139, F91 and N112) whose cultured samples were also *M. bovis* positive, indicating the presence of viable *M. bovis*. Deep sequencing of amplicon targets produced high numbers of total reads assigned to *Mycobacterium* genus, as shown at the top of Figure 5. The relative abundance (i.e., percentage of reads) of *M. bovis* DNA among all *Mycobacteria* spp. identified in the 20 goats is also shown in Figure 5. Results ranged from 0.8 to 97.5%. The three goat samples that were also culture-positive for *M. bovis* had a high percentage of *M. bovis*-specific reads, with goat C139 having 64.1%, F91 having 93.4%, and N112 with 68%. Interestingly, there were other goat samples with high percentages of *M. bovis* reads that did not have *M. bovis* detected in cultures, for example, goat O125 with 97.5%, and C126 with 65.8%. Raw samples from three goats with very low *M. bovis* abundance (E72 with 1.2%, G106 with 4.7%, I166 with 0.8%) were associated with having Ultra positive, but negative culture results. Although 20 goats had Ultra positive results, only 11 were confirmed to have *M. bovis* DNA using amplicon deep sequencing, and only 3 were considered *M. bovis* positive using culture.

The swab samples also contained a range of NTM species, which were identified based on the deep sequencing results. The relative abundances of different NTM species and *M. bovis* in the 20 Ultra positive goats are shown in Figure 5. The NTM species identified included *M. avium* complex, as well as *M. kansasii, M. fortuitum, M. goodii, M. flavescens, M. terrae*, and *M. virginiense* (Supplementary Table 2).

## 4 Discussion

This study aimed to determine whether *M. bovis* could be detected in nasal swabs from goats sharing pastures and water points with *M. bovis* infected cattle in KwaZulu-Natal, SA. Using mycobacterial culture and culture-independent techniques, 53 out of 157 were identified with matched *Mycobacterium* spp. These goat samples were further characterized using the Ultra as a rapid sensitive screening method, which resulted in 20 goat swabs suspected to contain MTBC DNA. Based on PCR results from DNA extracted from cultures, 3 of the 20 Ultra positive goat samples were identified to have viable *M. bovis* present in respiratory secretions. However, deep sequencing of PCR amplicons using DNA extracted directly from swabs detected an additional eight goats with *M. bovis* DNA, as well as the three culture positive goats. Based on the presence of *M. bovis* DNA in 11 goat nasal samples, it appeared that communal goats were exposed and possibly infected. This is not surprising since it was hypothesized that these goats were at risk since they shared grazing and water sources with *M. bovis* infected cattle, could have undetected goat to goat transmission, and a low likelihood of interaction with infected wildlife in nearby game parks, as has been previously observed (Ciaravino et al., 2021). Results also highlight the potential value of culture-independent detection methods for epidemiological investigations as well as an ancillary method for antemortem diagnosis of *M. bovis* in goats.

A large portion of the nasal swabs were positive for growth in MGIT cultures (78%; 123/157). This was expected since nasal swabs were likely to contain environmental contamination with NTMs and other organisms. Non-tuberculous mycobacteria are ubiquitous in the environment and studies have confirmed a high diversity of environmental NTMs in SA (Gcebe et al., 2013). This was likely why there was a high percentage (43%; 53/123) of positive cultures that contained matching mycobacterial species in both the culture isolate and DNA extracted directly from swabs. The observed diversity of NTMs identified suggests that these goats were exposed to environmental mycobacteria, many of which have been recently proven to confound *M. bovis* diagnosis in guinea pigs, especially when using tests based on host immune responses (Fernández-Veiga et al., 2023). Further studies are needed to investigate the association between presence of NTMs and potential cross-reactivity in *M. bovis* specific cell-mediated immunological tests in goats.

Although matched *Mycobacterium* spp. was identified in culture and directly from swabs in 53 goats, Sanger sequencing results could not distinguish NTM from MTBC. Therefore, the Ultra was used as a rapid, sensitive method to screen raw swabs for the presence of MTBC in these samples. One goat from the commercial stud herd was among the 20 Ultra positive goats. Since the herd history supported the assumption that commercial stud goats had no previous exposure to *M. bovis*, and further deep sequencing did not confirm the presence of *M. bovis*, this finding was most likely a false positive result. Although high bacterial loads of NTMs have been suspected as a cause of false positive Ultra results, one study determined that *M. abscessus, M. aurum, M. marinum, M. phlei*, and *M. smegmatis* do not lead to cross-reactivity (Huh et al., 2019). Furthermore, a positive Ultra result does not differentiate between dead or non-viable mycobacteria, or residual DNA within the sample, as has been observed in human patients (Miotto et al., 2012; Theron et al., 2018). Despite this, the Ultra’s diagnostic appeal, for use on animal specimens suspected of having MTBC infections, is increasing (Chakravorty et al., 2017), and has provided rapid detection of MTBC DNA in tissue and respiratory samples collected from infected African buffaloes (Clarke et al., 2021, 2022a), African elephants (Goosen et al., 2020), and white rhinoceros (Goosen et al., 2020). The positive Ultra results in the 19 communal goats were likely true positives, particularly since they share grazing and water sources with *M. bovis* infected cattle (Sichewo et al., 2020). Therefore, further analyses of Ultra positive samples were performed to speciate and confirm the presence of *M. bovis*.

Mycobacterial culture is the cornerstone for detecting viable MTBC in animal samples (Bernitz et al., 2021). Therefore, one of the swab aliquots from each goat was processed for mycobacterial culture to detect the presence of viable MTBC. Of the 20 Ultra positive goats, 3 were confirmed to have *M. bovis* growth in culture from their nasal swabs, based on genus-specific PCR results and confirmed by RD-PCR. All three samples were also Ultra positive, which shows promise as a screening tool to identify potential infected animals. Since it is known that nasal secretions may be paucibacillary and to overcome the suboptimal sensitivity of culture (Corner et al., 2012), the duration of culture for the goat nasal swabs was extended from the conventional 56 days to 365 days. The presence of viable *M. bovis,* in at least three samples suggests that these goats either were infected and shedding or had respiratory colonization. Although further testing would be required to differentiate between these conditions, this finding supports the hypothesis that communal goats in this area were likely exposed and may become infected with *M. bovis*.

There were eight additional goats identified with *M. bovis* DNA, based on amplicon deep sequencing. Since these were only detected using DNA extracted directly from swabs, it suggests that these samples were paucibacillary, in a non-culturable form, or represented residual *M. bovis* DNA. These results may be attributed to several factors. The number of viable bacteria in nasal samples can differ depending on the stage of disease and intermittent shedding (de la Rua-Domenech et al., 2006; de Souza Figueiredo et al., 2010), affecting the culture outcome. A sample’s viable bacterial load may also be reduced during the collection, handling and storage of samples (Corner et al., 2012; Goosen et al., 2022b). The culture of MTBC also involves a decontamination process, which can reduce the amount of viable MTBC (Burdz et al., 2003; Steingart et al., 2006; Madigan, 2012). Nasal swabs are expected to contain a high number of contaminating microbes, including environmental non-tuberculous mycobacteria, which may outcompete or inhibit growth of slow growing MTBC (Robbe-Austerman et al., 2013). This was supported by the high proportion of MGIT cultures with positive growth. Since the swab sample diluent was split for different analyses, it is also possible that the presence and number of MTBC bacilli varied between the aliquots. The most likely explanations for the discordant results between direct and culture detection were that MTBC were present in low numbers, had variable viability, and the high level of contamination inhibited growth of MTBC in culture. Therefore, MTBC DNA detection should employ more than one technique to increase confidence in results.

The relative abundance of *M. bovis* DNA, determined by amplicon deep sequencing, varied in the 11 positive goat samples, which is not surprising given the complex nature of the sample. The 3 *M. bovis* culture positive goats had higher numbers of *M. bovis-*specific reads (range 91,310–135,956). Six of the 11 goats had *M. bovis*-specific sequence reads >79,000, increasing confidence in these results, despite not finding viable bacilli by culture in some cases. However, we were unable to discern whether the *M. bovis* was inhaled from the environment vs. being secreted by an infected individual. Despite this limitation, this finding is important since it suggests *M. bovis* was present in this system. A study in humans suggested that presence of *M. tuberculosis* DNA in nasal swabs might precede development of pulmonary infection (Balcells et al., 2016). We speculated that goats with higher numbers of *M. bovis* reads might indicate that these goats were infected and shedding, vs. simple contamination since lower numbers of bacilli would likely be present in the environment (Santos et al., 2015). However, without ancillary testing, such as *M. bovis* specific immunological responses in these goats, it is difficult to discern the source of the mycobacteria. Further studies should explore the association between numbers of sequence reads and likelihood of infection.

The use of whole genome and targeted next-generation sequencing (tNGS) for diagnosis and detection of drug resistance in *Mycobacterium tuberculosis* in clinical samples has grown exponentially in recent years (Nimmo et al., 2019; Cabibbe et al., 2020). The advantages are rapid culture-independent results to inform patient care. In addition, this approach has shown that there is greater genomic diversity in sputum than sequences derived from culture (Nimmo et al., 2019). Similar to this goat study, Kambli et al. (2021) screened sputum with Ultra and then used tNGS to diagnose patients and determine drug resistance profiles (Kambli et al., 2021). Bacterial load, as assessed by Ultra, appeared to correlate with *hsp65* gene coverage depth. In livestock and wildlife, PCR-based tools have been used with oronasal swabs to detect *Mycobacterium bovis* infection and shedding (McCorry et al., 2005; Clarke et al., 2022b). In naturally infected wild boar, nasal shedding of MTBC DNA was detected in 40.8% of TB-affected animals and 73.6% of these had generalized TB lesions in head lymph nodes and lungs (Risco et al., 2019). Therefore, it is likely that at least some of the goats with positive nasal swabs were infected, although this could not be confirmed due to limitations of the tests performed.

This study had several limitations, including the inability to confirm infection/disease using antemortem tests to detect *M. bovis-*specific host immune responses, or postmortem techniques including mycobacterial culture and histopathology. Although three goats did have *M. bovis* isolated by culture, it was only after an extended (365 days) period of incubation and was likely a very paucibacillary sample, which may not represent true infection. The presence of NTMs in the complex samples could have also obscured the presence of *M. bovis* by rapid overgrowth. Most positive goats had only *M. bovis* DNA detected and this could represent contamination from environmental sources or residual DNA rather than infection. Variability between PCR amplification and culturability among different mycobacteria species may also contribute to some of the discrepancies observed. Furthermore, attempts to obtain further genomic data for the *M. bovis* positive samples through both culture and raw aliquots, were unsuccessful, with spoligotyping returning indistinguishable patterns. Additional techniques will be pursued to characterize these isolates. In addition, the process for identifying *M. bovis* DNA in these 11 goats required several labor-intensive and costly steps, including multiple PCRs and deep sequencing. Therefore, although this approach may be valuable for research investigations and routine surveillance in developed countries, at the moment, it is a bit more challenging for developing countries.

## 5 Conclusion

The detection of viable *M. bovis* and DNA in goat nasal passages provides evidence that goats were exposed and potentially infected. Although one dairy goat had an Ultra positive result, the majority of goats with *M. bovis* DNA detected were from communal herds, which suggests that there may be a greater risk of infection in goats that share an environment with infected cattle. The Ultra appeared to be a useful screening tool to detect MTBC and select nasal swabs for additional analyses. The genus-specific PCR with amplicon Sanger sequencing and RD-PCR could identify animals with higher abundance of *M. bovis,* but miss animals with lower abundance, which could be found with targeted deep sequencing. This culture-independent approach has promise for improved detection of *M. bovis* in paucibacillary samples from goats.

## Data availability statement

The datasets presented in this study can be found in an online repository available at: https://www.ebi.ac.uk/ena/browser/search under project reference number PRJEB70955. Moreover, samples-, experiment- and run accession numbers can be found under Supplementary material.

## Ethics statement

The animal studies were approved by Stellenbosch University Animal Care and Use Committee (ACU‐2020‐14560) and the Department of Agriculture, Land Reform, and Rural Development [12/11/1/7/2 (16045S)]. The studies were conducted in accordance with the local legislation and institutional requirements. Written informed consent was obtained from the owners for the participation of their animals in this study.

## Author contributions

DC: Formal Analysis, Investigation, Methodology, Visualization, Writing – original draft. CC: Investigation, Methodology, Resources, Writing – review & editing. TK: Data curation, Methodology, Writing – review & editing. RW: Formal Analysis, Methodology, Supervision, Writing – review & editing. CW: Data curation, Formal Analysis, Investigation, Methodology, Writing – review & editing. MM: Conceptualization, Data curation, Formal Analysis, Funding acquisition, Investigation, Methodology, Project administration, Resources, Supervision, Visualization, Writing – original draft, Writing – review & editing. WG: Conceptualization, Data curation, Formal Analysis, Funding acquisition, Investigation, Methodology, Project administration, Resources, Supervision, Validation, Visualization, Writing – original draft, Writing – review & editing.

## Footnotes

^1^ https://view.qiime2.org/

## References

[ref1] Adékambi T. Colson P. Drancourt M. (2003). *rpoB*-based identification of nonpigmented and late-pigmenting rapidly growing mycobacteria. J. Clin. Microbiol. 41, 5699–5708. doi: 10.1128/JCM.41.12.5699-5708.2003, PMID: 14662964 PMC308974

[ref2] Arnot L. F. Michel A. (2020). Challenges for controlling bovine tuberculosis in South Africa. Onderstepoort J. Vet. Res. 87, e1–e8. doi: 10.4102/ojvr.v87i1.1690, PMID: 32129639 PMC7059242

[ref3] Balcells M. E. Huilcamán M. Peña C. Castillo C. Carvajal C. Scioscia N.. (2016). *M. tuberculosis* DNA detection in nasopharyngeal mucosa can precede tuberculosis development in contacts. Int. J. Tuberc. Lung Dis. 20, 848–852. doi: 10.5588/ijtld.15.0872, PMID: 27155192

[ref4] Bernitz N. Kerr T. J. Goosen W. J. Chileshe J. Higgitt R. L. Roos E. O.. (2021). Review of diagnostic tests for detection of *Mycobacterium bovis* infection in south African wildlife. Front. Vet. Sci. 8:697. doi: 10.3389/fvets.2021.588697, PMID: 33585615 PMC7876456

[ref5] Bezos J. Casal C. Díez-Delgado I. Romero B. Liandris E. Álvarez J.. (2015). Goats challenged with different members of the *Mycobacterium tuberculosis* complex display different clinical pictures. Vet. Immunol. Immunopathol. 167, 185–189. doi: 10.1016/j.vetimm.2015.07.009, PMID: 26235598

[ref6] Bolaños C. A. D. Paula C. L. Guerra S. T. Franco M. M. J. Ribeiro M. G. (2017). Diagnosis of mycobacteria in bovine milk: an overview. Rev. Inst. Med. Trop. Sao Paulo 59:e40. doi: 10.1590/S1678-9946201759040 28591268 PMC5466425

[ref7] Borham M. Oreiby A. El-Gedawy A. Hegazy Y. Khalifa H. O. Al-Gaabary M.. (2022). Review on bovine tuberculosis: an emerging disease associated with multidrug-resistant *Mycobacterium* species. Pathogens 11:715. doi: 10.3390/pathogens11070715, PMID: 35889961 PMC9320398

[ref8] Brückner G. K. Vosloo W. Du Plessis B. J. A. Kloeck P. E. L. G. Connoway L. Ekron M. D.. (2002). Foot and mouth disease: the experience of South Africa. Rev. Sci. Tech. 21, 751–764. doi: 10.20506/rst.21.3.1368 12523712

[ref9] Burdz T. V. N. Wolfe J. Kabani A. (2003). Evaluation of sputum decontamination methods for *Mycobacterium tuberculosis* using viable colony counts and flow cytometry. Diagn. Microbiol. Infect. Dis. 47, 503–509. doi: 10.1016/s0732-8893(03)00138-x, PMID: 14596969

[ref10] Cabibbe A. M. Spitaleri A. Battaglia S. Colman R. E. Suresh A. Uplekar S.. (2020). Application of targeted next-generation sequencing assay on a portable sequencing platform for culture-free detection of drug-resistant tuberculosis from clinical samples. J. Clin. Microbiol. 58, e00632–e00620. doi: 10.1128/JCM.00632-20, PMID: 32727827 PMC7512157

[ref11] Chakravorty S. Simmons A. M. Rowneki M. Parmar H. Cao Y. Ryan J.. (2017). The new Xpert MTB/RIF ultra: improving detection of *Mycobacterium tuberculosis* and resistance to rifampin in an assay suitable for point-of-care testing. MBio 8, e00812–e00817. doi: 10.1128/mBio.00812-17 28851844 PMC5574709

[ref12] Ciaravino G. Vidal E. Cortey M. Martín M. Sanz A. Mercader I.. (2021). Phylogenetic relationships investigation of *Mycobacterium caprae* strains from sympatric wild boar and goats based on whole genome sequencing. Transbound. Emerg. Dis. 68, 1476–1486. doi: 10.1111/tbed.13816, PMID: 32888386 PMC8246549

[ref13] Clarke C. Cooper D. V. Miller M. A. Goosen W. J. (2022a). Detection of *Mycobacterium tuberculosis* complex DNA in oronasal swabs from infected African buffaloes (*Syncerus caffer*). Sci. Rep. 12, 1834–1836. doi: 10.1038/s41598-022-05982-6, PMID: 35115633 PMC8813999

[ref14] Clarke C. Kerr T. J. Warren R. M. Kleynhans L. Miller M. A. Goosen W. J. (2022b). Identification and characterisation of nontuberculous mycobacteria in African buffaloes (*Syncerus caffer*), South Africa. Microorganisms 10:1861. doi: 10.3390/microorganisms10091861, PMID: 36144463 PMC9503067

[ref15] Clarke C. Smith K. Goldswain S. J. Helm C. Cooper D. V. Kerr T. J.. (2021). Novel molecular transport medium used in combination with Xpert MTB/RIF ultra provides rapid detection of *Mycobacterium bovis* in African buffaloes. Sci. Rep. 11, 7061–7066. doi: 10.1038/s41598-021-86682-5, PMID: 33782515 PMC8007588

[ref16] Cooke D. M. Goosen W. J. Burgess T. Witte C. Miller M. A. (2023). *Mycobacterium tuberculosis* complex detection in rural goat herds in South Africa using Bayesian latent class analysis. Vet. Immunol. Immunopathol. 257:110559. doi: 10.1016/j.vetimm.2023.110559, PMID: 36739737

[ref17] Corner L. A. L. Gormley E. Pfeiffer D. U. (2012). Primary isolation of *Mycobacterium bovis* from bovine tissues: conditions for maximising the number of positive cultures. Vet. Microbiol. 156, 162–171. doi: 10.1016/j.vetmic.2011.10.016, PMID: 22074859

[ref18] de Boer A. S. Blommerde B. de Haas P. E. W. Sebek M. M. G. G. Lambregts-van Weezenbeek K. S. B. Dessens M.. (2002). False-positive *Mycobacterium tuberculosis* cultures in 44 laboratories in the Netherlands (1993 to 2000): incidence, risk factors, and consequences. J. Clin. Microbiol. 40, 4004–4009. doi: 10.1128/JCM.40.11.4004-4009.2002, PMID: 12409366 PMC139647

[ref19] de la Rua-Domenech R. Goodchild A. T. Vordermeier H. M. Hewinson R. G. Christiansen K. H. Clifton-Hadley R. S. (2006). Ante mortem diagnosis of tuberculosis in cattle: a review of the tuberculin tests, gamma-interferon assay, and other ancillary diagnostic techniques. Res. Vet. Sci. 81, 190–210. doi: 10.1016/j.rvsc.2005.11.005, PMID: 16513150

[ref20] de Souza Figueiredo E. E. Carvalho R. C. T. Silvestre F. G. Lilenbaum W. Fonseca L. S. Silva J. T.. (2010). Detection of *Mycobacterium bovis* DNA in nasal swabs from tuberculous cattle by a multiplex PCR. Braz. J. Microbiol. 41, 386–390. doi: 10.1590/S1517-838220100002000020, PMID: 24031509 PMC3768681

[ref21] Deggim-Messmer V. Bloemberg G. V. Ritter C. Voit A. Hömke R. Keller P. M.. (2016). Diagnostic molecular mycobacteriology in regions with low tuberculosis Endemicity: combining real-time PCR assays for detection of multiple mycobacterial pathogens with line probe assays for identification of resistance mutations. EBioMedicine 9, 228–237. doi: 10.1016/j.ebiom.2016.06.016, PMID: 27333026 PMC4972562

[ref22] Department of Agriculture, Forestry and Fisheries of SA. (2016). Bovine tuberculosis manual. Available at: https://nahf.co.za/updated-tuberculosis-manual/ (Accessed 7 November 2023).

[ref001] Department of Agriculture, Forestry and Fisheries of SA. (2017). Abstract of agricultural statistics 2015. Available at: http://webapps.daff.gov.za/AmisAdmin/upload/2017%20Abstract%20%20of%20Agricultural%20Statistics.pdf (Accessed 3 November 2023).

[ref23] Department of Agriculture, Land Reform and Rural Development. (2018). Tuberculosis testing in sheep and goat’s manual. Available at: https://nahf.co.za/tuberculosis-testing-in-sheep-and-goats-2018-11-29/ (Accessed 7 November 2023).

[ref24] Deurenberg R. H. Bathoorn E. Chlebowicz M. A. Couto N. Ferdous M. García-Cobos S.. (2017). Application of next generation sequencing in clinical microbiology and infection prevention. J. Biotechnol. 243, 16–24. doi: 10.1016/j.jbiotec.2016.12.022, PMID: 28042011

[ref25] Falkinham J. O. (2021). Ecology of nontuberculous mycobacteria. Microorganisms 9:2262. doi: 10.3390/microorganisms9112262 34835388 PMC8625734

[ref26] Fernández-Veiga L. Fuertes M. Geijo M. V. Pérez de Val B. Vidal E. Michelet L.. (2023). Differences in skin test reactions to official and defined antigens in guinea pigs exposed to non-tuberculous and tuberculous bacteria. Sci. Rep. 13:2936. doi: 10.1038/s41598-023-30147-4, PMID: 36806813 PMC9941491

[ref27] Gcebe N. Rutten V. Gey van Pittius N. C. Michel A. (2013). Prevalence and distribution of non-tuberculous mycobacteria (NTM) in cattle, African buffaloes (*Syncerus caffer*) and their environments in South Africa. Transbound. Emerg. Dis. 60, 74–84. doi: 10.1111/tbed.12133 24171852

[ref28] Ghodbane R. Raoult D. Drancourt M. (2014). Dramatic reduction of culture time of *Mycobacterium tuberculosis*. Sci. Rep. 4:4236. doi: 10.1038/srep04236, PMID: 24577292 PMC3937792

[ref29] Goosen W. J. Clarke C. Kleynhans L. Kerr T. J. Buss P. Miller M. A. (2022a). Culture-independent PCR detection and differentiation of *mycobacteria spp*. in antemortem respiratory samples from African elephants (*Loxodonta africana*) and rhinoceros (*Ceratotherium simum, Diceros bicornis*) in South Africa. Pathogens 11:709. doi: 10.3390/pathogens11060709, PMID: 35745564 PMC9230505

[ref30] Goosen W. J. Kerr T. J. Kleynhans L. Warren R. M. van Helden P. D. Persing D. H.. (2020). The Xpert MTB/RIF ultra assay detects *Mycobacterium tuberculosis* complex DNA in white rhinoceros (*Ceratotherium simum*) and African elephants (*Loxodonta africana*). Sci. Rep. 10:14482. doi: 10.1038/s41598-020-71568-9, PMID: 32879401 PMC7468236

[ref31] Goosen W. J. Kleynhans L. Kerr T. J. van Helden P. D. Buss P. Warren R. M.. (2022b). Improved detection of *Mycobacterium tuberculosis* and *M. bovis* in African wildlife samples using cationic peptide decontamination and mycobacterial culture supplementation. J. Vet. Diagn. Invest. 34, 61–67. doi: 10.1177/10406387211044192, PMID: 34510986 PMC8688974

[ref32] Huh H. J. Song D. J. Ki C. S. Lee N. Y. (2019). Is cross-reactivity with nontuberculous mycobacteria a systematic problem in the Xpert MTB/RIF assay? Tuberc. Respir. Dis. 82, 88–89. doi: 10.4046/trd.2018.0075, PMID: 30574692 PMC6304324

[ref33] Jung Y. J. Kim J.-Y. Song D. J. Koh W.-J. Huh H. J. Ki C.-S.. (2016). Evaluation of three real-time PCR assays for differential identification of *Mycobacterium tuberculosis* complex and nontuberculous mycobacteria species in liquid culture media. Diagn. Microbiol. Infect. Dis. 85, 186–191. doi: 10.1016/j.diagmicrobio.2016.03.014, PMID: 27105774

[ref34] Kambli P. Ajbani K. Kazi M. Sadani M. Naik S. Shetty A.. (2021). Targeted next generation sequencing directly from sputum for comprehensive genetic information on drug resistant *Mycobacterium tuberculosis*. Tuberculosis 127:102051. doi: 10.1016/j.tube.2021.102051, PMID: 33450448

[ref35] Kamerbeek J. Schouls L. Kolk A. van Agterveld M. van Soolingen D. Kuijper S.. (1997). Simultaneous detection and strain differentiation of *Mycobacterium tuberculosis* for diagnosis and epidemiology. J. Clin. Microbiol. 35, 907–914. doi: 10.1128/jcm.35.4.907-914.1997, PMID: 9157152 PMC229700

[ref36] Lee R. S. Proulx J.-F. McIntosh F. Behr M. A. Hanage W. P. (2020). Previously undetected super-spreading of *Mycobacterium tuberculosis* revealed by deep sequencing. Elife 9:e53245. doi: 10.7554/eLife.53245, PMID: 32014110 PMC7012596

[ref37] Madigan G. (2012). Evaluation of different methods for the detection of *Mycobacterium bovis* in lymph node tissue. Thesis of Master of Science, National University of Ireland Maynooth. 37.

[ref38] McCorry T. Whelan A. O. Welsh M. D. McNair J. Walton E. Bryson D. G.. (2005). Shedding of *Mycobacterium bovis* in the nasal mucus of cattle infected experimentally with tuberculosis by the intranasal and intratracheal routes. Vet. Rec. 157, 613–618. doi: 10.1136/vr.157.20.613, PMID: 16284329

[ref39] Michel A. L. Cooper D. Jooste J. de Klerk L.-M. Jolles A. (2011). Approaches towards optimizing the gamma interferon assay for diagnosing *Mycobacterium bovis* infection in African buffalo (*Syncerus caffer*). Prev. Vet. Med. 98, 142–151. doi: 10.1016/j.prevetmed.2010.10.016 21122932

[ref40] Miotto P. Bigoni S. Migliori G. B. Matteelli A. Cirillo D. M. (2012). Early tuberculosis treatment monitoring by Xpert® MTB/RIF. Eur. Respir. J. 39, 1269–1271. doi: 10.1183/09031936.00124711, PMID: 22547737

[ref41] More S. J. (2019). Can bovine TB be eradicated from the Republic of Ireland? Could this be achieved by 2030? Ir. Vet. J. 72:3. doi: 10.1186/s13620-019-0140-x, PMID: 31057791 PMC6485114

[ref42] Mostowy S. Inwald J. Gordon S. Martin C. Warren R. Kremer K.. (2005). Revisiting the evolution of *Mycobacterium bovis*. J. Bacteriol. 187, 6386–6395. doi: 10.1128/JB.187.18.6386-6395.2005 16159772 PMC1236643

[ref43] Nimmo C. Shaw L. P. Doyle R. Williams R. Brien K. Burgess C.. (2019). Whole genome sequencing *Mycobacterium tuberculosis* directly from sputum identifies more genetic diversity than sequencing from culture. BMC Genomics 20:389. doi: 10.1186/s12864-019-5782-2, PMID: 31109296 PMC6528373

[ref44] Nyoni G., (2019). Questionnaire-based study to determine the state of tuberculosis testing in goats in South Africa. (MSc (Tropical Animal Health) mini dissertation), University of Pretoria. Available at: http://hdl.handle.net/2263/76754 (Accessed 20 January 2024).

[ref45] Palmer M. V. Thacker T. C. Waters W. R. Gortázar C. Corner L. A. L. (2012). *Mycobacterium bovis*: a model pathogen at the interface of livestock, wildlife, and humans. Vet Med Int 2012:236205. doi: 10.1155/2012/236205, PMID: 22737588 PMC3377356

[ref46] Palmer M. V. Waters W. R. (2011). Bovine tuberculosis and the establishment of an eradication program in the United States: role of veterinarians. Vet Med Int 2011, 1–12. doi: 10.4061/2011/816345, PMID: 21647341 PMC3103864

[ref47] Pesciaroli M. Alvarez J. Boniotti M. B. Cagiola M. Di Marco V. Marianelli C.. (2014). Tuberculosis in domestic animal species. Res. Vet. Sci. 97, S78–S85. doi: 10.1016/j.rvsc.2014.05.015 25151859

[ref48] Quintas H. Reis J. Pires I. Alegria N. (2010). Tuberculosis in goats. Vet. Rec. 166, 437–438. doi: 10.1136/vr.c1678 20364017

[ref49] Renwick A. R. White P. C. Bengis R. G. (2007). Bovine tuberculosis in southern African wildlife: a multi-species host-pathogen system. Epidemiol. Infect. 135, 529–540. doi: 10.1017/S0950268806007205, PMID: 16959052 PMC2870607

[ref50] Reviriego Gordejo F. J. Vermeersch J. P., (2006). Towards eradication of bovine tuberculosis in the European Union. Veterinary microbiology, In: *4th International Conference on Mycobacterium bovis.* 112, 101–109. 10.1016/j.vetmic.2005.11.034 16388921

[ref51] Risco D. Martínez R. Bravo M. Fernández Llario P. Cerrato R. Garcia-Jiménez W. L.. (2019). Nasal shedding of *Mycobacterium tuberculosis* in wild boar is related to generalized tuberculosis and concomitant infections. Vet. Rec. 185:629. doi: 10.1136/vr.105511, PMID: 31515441

[ref52] Robbe-Austerman S. Bravo D. M. Harris B. (2013). Comparison of the MGIT 960, BACTEC 460 TB and solid media for isolation of *Mycobacterium bovis* in United States veterinary specimens. BMC Vet. Res. 9:74. doi: 10.1186/1746-6148-9-74, PMID: 23578209 PMC3637409

[ref53] Rodríguez E. Sánchez L. P. Pérez S. Herrera L. Jiménez M. S. Samper S.. (2009). Human tuberculosis due to *Mycobacterium bovis* and *M. caprae* in Spain, 2004–2007. Int. J. Tuberc. Lung Dis. 13, 1536–1541. Available at: https://pubmed.ncbi.nlm.nih.gov/19919773/. 19919773

[ref54] Santos N. Santos C. Valente T. Gortázar C. Almeida V. Correia-Neves M. (2015). Widespread environmental contamination with *Mycobacterium tuberculosis* complex revealed by a molecular detection protocol. PLoS One 10:e0142079. doi: 10.1371/journal.pone.0142079, PMID: 26561038 PMC4641585

[ref55] Shi W. Hu Y. Zheng X. Ning Z. Wu M. Xia F.. (2019). Longitudinal profiling of gut microbiome among tuberculosis patients under anti-tuberculosis treatment in China: protocol of a prospective cohort study. BMC Pulm. Med. 19:211. doi: 10.1186/s12890-019-0981-9, PMID: 31711450 PMC6849301

[ref56] Sichewo P. R. Etter E. M. C. Michel A. L. (2020). Wildlife-cattle interactions emerge as drivers of bovine tuberculosis in traditionally farmed cattle. Prev. Vet. Med. 174:104847. doi: 10.1016/j.prevetmed.2019.104847, PMID: 31786405

[ref57] Steingart K. R. Ng V. Henry M. Hopewell P. C. Ramsay A. Cunningham J.. (2006). Sputum processing methods to improve the sensitivity of smear microscopy for tuberculosis: a systematic review. Lancet Infect. Dis. 6, 664–674. doi: 10.1016/S1473-3099(06)70602-8 17008175

[ref58] Theron G. Venter R. Smith L. Esmail A. Randall P. Sood V.. (2018). False-positive Xpert MTB/RIF results in retested patients with previous tuberculosis: frequency, profile, and prospective clinical outcomes. J. Clin. Microbiol. 56:17. doi: 10.1128/JCM.01696-17, PMID: 29305538 PMC5824043

[ref59] Vordermeier H. M. Brown J. Cockle P. J. Franken W. P. J. Drijfhout J. W. Arend S. M.. (2007). Assessment of cross-reactivity between *Mycobacterium bovis* and *M. kansasii ESAT-6* and *CFP-10* at the T-cell epitope level. Clin. Vaccine Immunol. 14, 1203–1209. doi: 10.1128/CVI.00116-07, PMID: 17671227 PMC2043317

[ref60] Warren R. M. Gey van Pittius N. C. Barnard M. Hesseling A. Engelke E. de Kock M.. (2006). Differentiation of *Mycobacterium tuberculosis* complex by PCR amplification of genomic regions of difference. Int. J. Tuberc. Lung Dis. 10, 818–822. PMID: 16850559

[ref61] Welsh M. D. Cunningham R. T. Corbett D. M. Girvin R. M. McNair J. Skuce R. A.. (2005). Influence of pathological progression on the balance between cellular and humoral immune responses in bovine tuberculosis. Immunology 114. doi: 10.1111/j.1365-2567.2004.02003 PMC1782060 15606800

[ref62] World Health Organization. (2017). Roadmap for zoonotic tuberculosis. Available at: https://www.who.int/publications/i/item/9789241513043. (Accessed 6 June 2023).

[ref63] World Health Organization. (2020). Global Tuberculosis report 2020, Geneva, Switzerland. Available at: https://www.who.int/publications/i/item/9789240013131 (Accessed 20 January 2024).

[ref64] World Organization for Animal Health. (2023). Available at: https://www.woah.org/en/what-we-do/animal-health-and-welfare/animal-diseases/old-classification-of-diseases-notifiable-to-the-oie-list-b/ (Accessed 5 June 2023).

